# Xanthogranulomatous pyelonephritis in a patient with polycystic kidney disease without underlying risk factors: a case report

**DOI:** 10.3389/fmed.2024.1419965

**Published:** 2024-08-09

**Authors:** Yoomee Kang, Tae Won Lee, Eunjin Bae, Ha Nee Jang, Sehyun Jung, Seunghye Lee, Se-Ho Chang, Dong Jun Park

**Affiliations:** ^1^Department of Internal Medicine, Gyeongsang National University Changwon Hospital, Changwon, Republic of Korea; ^2^Department of Internal Medicine, Gyeongsang National University College of Medicine, Jinju, Republic of Korea; ^3^Institute of Health Science, Gyeongsang National University, Jinju, Republic of Korea; ^4^Department of Internal Medicine, Gyeongsang National University Hospital, Jinju, Republic of Korea

**Keywords:** xanthogranulomatous pyelonephritis, polycystic kidney disease, fever, abdominal pain, renal mass

## Abstract

Xanthogranulomatous pyelonephritis (XGP) is an extremely rare, chronic granulomatous inflammatory condition thought to arise secondary to a combination of obstruction, recurrent bacterial infection and an incomplete immune response although the etiology of XGP is more complex. We would like to report a case of XGP occurring in a patient with polycystic kidney disease (PCKD), which has not been previously documented in etiology. A 29-year-old woman presented to our hospital with right upper quadrant pain for 5 days. She had experienced a low-grade fever, generalized weakness, and myalgia throughout her body for 2 weeks. She had no history of renal stones or recurrent UTIs. Contrast-enhanced CT revealed a well-enhancing large septated cystic mass in the right kidney and numerous cysts in the liver and both kidneys. Open right radical nephrectomy was performed due to the suspicion of renal cell carcinoma, as there was no response to antibiotics over 7 days. Gross specimen demonstrated architectural distortion due to xanthomatous nodules and a dilated pelvico-calyceal system filled with pus and blood. Microscopic examination revealed infiltration of neutrophils and lipid-laden macrophages. The patient is currently being followed up in the outpatient clinic without recurrence of XGP. This is the first reported case of XGP in a patient with underlying PCKD. Physicians should consider PCKD as a potential underlying cause of XGP.

## Introduction

Xanthogranulomatous pyelonephritis (XGP) is a rare form of chronic granulomatous inflammation, characterized by the destruction of renal parenchyma and its replacement by solid sheets of lipid-laden macrophages, resulting in a non-functioning kidney (1). The prevalence of XGP varies from 0.6 to 1% of all cases of renal infections, with an incidence of 1.4 cases per 100,000 population per year. While the disease can occur in all age groups, it is more common in women than in men, typically affecting individuals in their fifth and sixth decades of life. There is no racial predilection (2–5). XGP is frequently referred to as a pseudotumor because the enlarged kidney resembles a tumor and leads to local invasion and destruction (1–5).

The term “xantho” originates from the infiltration of lipid-laden macrophages, which appear yellow in pathological sections. XGP typically arises in the setting of obstructive uropathy, nephrolithiasis, or urinary tract infections (UTIs) (1). The current concept of XGP was established by Osterlind (6) in 1944, although it was initially described by Schlagenhaufer (7) in 1916. While the disease typically manifests diffusely, it can sometimes be focal. The diagnosis of XGP is challenging due to its nonspecific findings, often progressing insidiously until the development of late-stage extrarenal sequelae (1). Preoperative diagnosis is also difficult because radiological imaging features can overlap with various other conditions, including abscess, lymphomas, angiolipomas, leiomyosarcomas, Wilms tumor, renal cell carcinoma (RCC), transitional cell carcinoma, renal tuberculosis, and malakoplakia (1, 2, 8). If left untreated, the disease can progress to complications necessitating nephrectomy. We present the first reported case of XGP occurring in a patient with polycystic kidney disease (PCKD) without major risk factors such as obstructive uropathy or recurrent UTIs.

## Case report

A 29-year-old Korean woman presented to our hospital with worsening pain in her right upper quadrant (RUQ) for 5 days. She had experienced a mild fever, generalized weakness, and myalgia throughout her body for 2 weeks, without any urinary symptoms such as frequency, dysuria, or urgency. Initially, she attributed her symptoms to a cold and took acetaminophen. However, the pain worsened and the fever persisted. She had no significant medical history, such as renal stones or recurrent UTIs, and denied any genetic diseases, including PCKD. She also denied any recent travel or use of drugs or herbal medications except for acetaminophen. Upon physical examination, she appeared acutely ill.

Her vital signs were as follows: blood pressure (BP), 130/80 mmHg; body temperature (BT), 38.5°C; heart rate (HR), 68 beats per min; and respiratory rate (RR), 20 breaths per min. She was alert and oriented with no abnormalities noted on neurological examination. Conjunctival pallor was present, but sclerae were not icteric. No palpable cervical lymphadenopathy or skin color changes were observed. Lung auscultation revealed no wheeze or murmurs. A soft, tender mass was palpable in the RUQ, and there was tenderness on percussion over the right costovertebral angle. There was no pitting edema noted in her lower extremities.

Her initial laboratory findings were as follows: leukocyte count, 12.09 (range: 4.0–10.0) × 10^9^/L; neutrophils, 80%; lymphocytes, 10.9%; monocytes, 5.9%; hemoglobin, 8.3 (range: 12–16) g/dL; platelet count, 167 (range: 130–400) × 10^9^/L; blood urea nitrogen, 6 (range: 8.0–20.0) mg/dL; creatinine, 0.5 (range: 0.51–0.95) mg/dL; total protein, 6.9 (range: 6.6–8.7) g/dL; albumin, 3.3 (range: 3.5–5.2) g/dL; total cholesterol, 105 (range: 120–200) mg/dL; aspartate transaminase, 18 (range: 1–37) U/L; alanine transaminase, 14 (1–37) U/L; glucose, 103 (range: 70–110) mg/dL; sodium, 133 (range: 135–145) mmol/L; potassium, 3.4 (range: 3.4–5.1) mmol/L; chloride, 97 (range: 98–110) mmol/L; prothrombin time, 13.2 (range: 11.9–14.3) s; aPTT, 35.2 (range: 29.1–43.5) s; and C-reactive protein, 131 (range: 0–3) mg/L. Her serum iron profile was as follows: iron, 13 (normal range: 60–180) μg/dL; total iron-binding capacity, 294 (range: 230–430) μg/dL; transferrin saturation, 4% (range: 20–55%); and ferritin, 81.97 (range: 13–150) ng/mL. Urinalysis revealed specific gravity of 1.010, no proteins or blood, and 3+ white blood cells. Microscopy revealed numerous white blood cells, whereas urine smear did not detect any bacteria. Furthermore, urine and blood cultures were negative.

**Figure 1:**
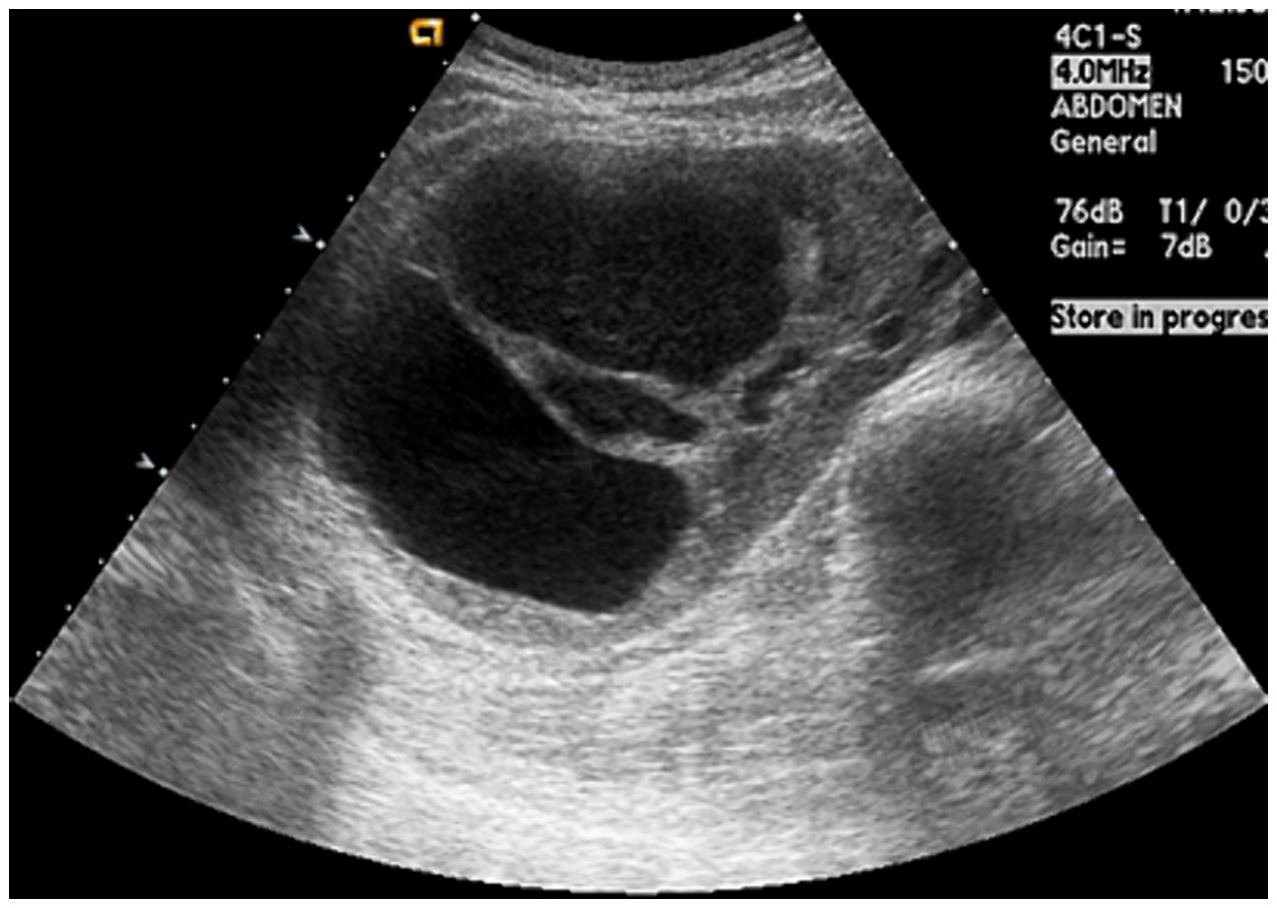
Ultrasonogaphy showing variable-sized numerous cysts and 8.8 cm multi-septated cystic mass on mid-lower pole of the right kidney.

**Figure 2:**
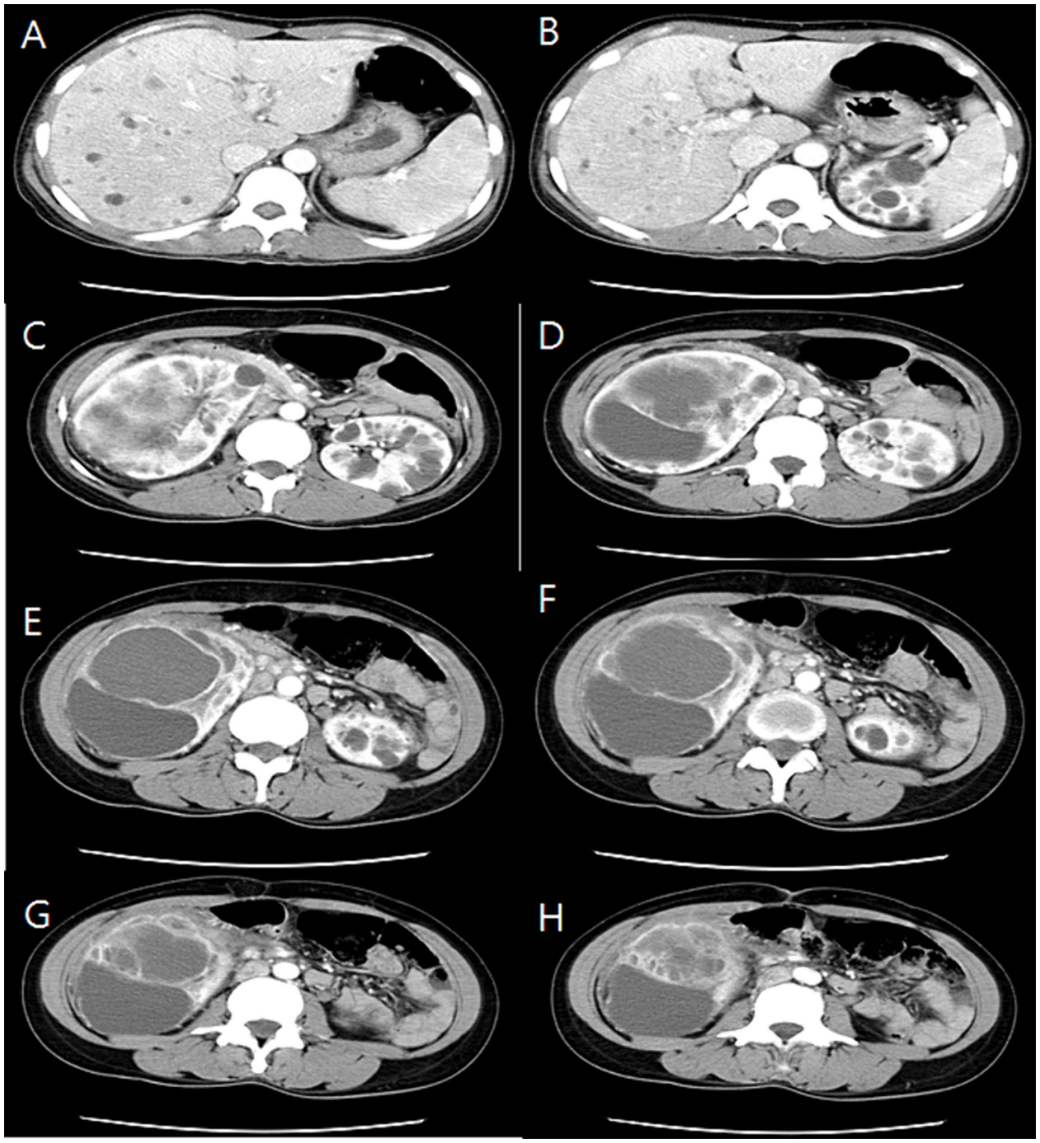
Serial section of computed tomography (CT) showing numeral cysts on liver **(A,B)** and upper pole of both kidneys **(C,D)** and 10 cm sized huge cystic mass with well-enhancing wall from mid-lower pole of right kidney **(E–H)**.

**Figure 3:**
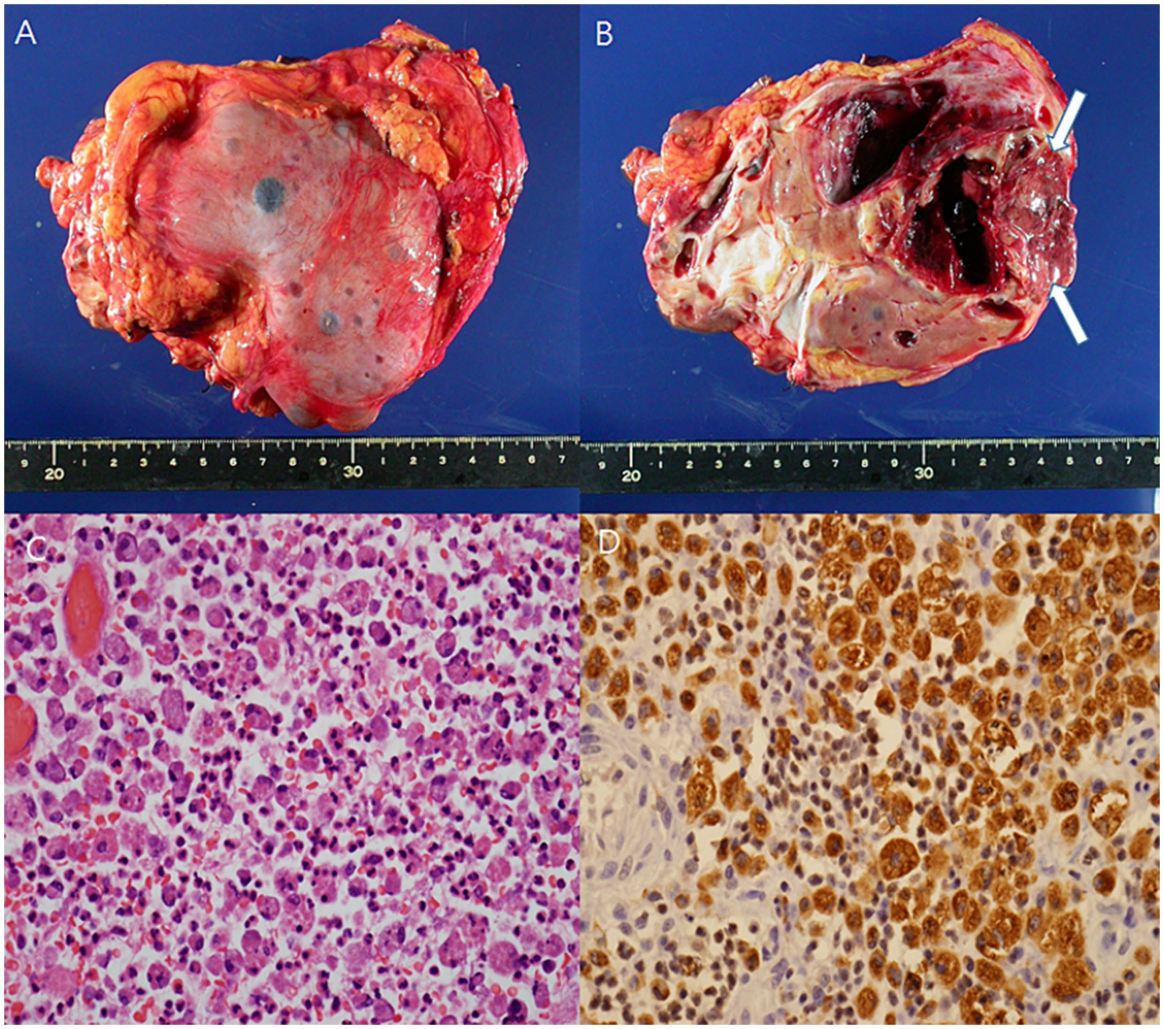
The gross specimen showed distortion of the architecture by xanthomatous nodule (arrow), and dilated pelvi-calyceal system filled with pus and blood **(A,B)**. Microscopic finding revealed infiltration of neutrophils and lipid-laden macrophages **(C)** (H/E, x400). Lipid-laden macrophage was diffusely stained for CD68 **(D)** (x400).

The initial kidney ultrasonography (USG) performed on admission day revealed variable sized renal cysts on both kidneys and an 8.8 cm multiseptate cystic mass on the mid-lower pole of the right kidney (Figure 1). Empirical treatment with intravenous ciprofloxacin was initiated under the suspicion of renal abscess, but the patient’s RUQ pain and fever persisted despite 3 days of antibiotic therapy. Her vital sings and significant laboratory tests measured on the third day of her hospitalization were as follows: BP, 120/70 mmHg; BT, 38.9°C; HR, 80 beats per min; and RR, 20 breaths per min and leukocyte count, 15.12 (range: 4.0–10.0) × 10^9^/L; neutrophils, 82%; lymphocytes, 11%; monocytes, 4.2%; hemoglobin, 8.1 (range: 12–16) g/dL; platelet count, 123 (range: 130–400) × 10^9^/L; blood urea nitrogen, 12 (range: 8.0–20.0) mg/dL; creatinine, 0.5 (range: 0.51–0.95) mg/dL; total protein, 6.9 (range: 6.6–8.7) g/dL; albumin, 3.1 (range: 3.5–5.2) g/dL; and C-reactive protein, 101 (range: 0–3) mg/L. Contrast-enhanced CT performed on the third day of hospitalization revealed multiple cysts in the liver and both kidneys, as well as a well-enhancing septate cystic mass measuring approximately 10 cm in length on the right kidney. Mass was limited to the kidney and there was no lymph node enlargement, invasion into major veins or perinephric tissues, and distant metastasis (T2a, N0, M0, stage II) (Figure 2). Open radical nephrectomy was performed on the seventh day after admission based on the presumed diagnosis of cystic RCC due to persistent clinical symptoms and signs. Upon sectioning the kidney, distortion of the normal architecture by xanthomatous nodules and dilated pelvicocalyces filled with pus and blood were observed (Figure 2). Microscopic examination revealed numerous lipid-laden CD68-positive macrophages (Figure 3). *Escherichia coli* was cultured from tissue samples obtained during the operation. Additional intravenous ciprofloxacin was administered starting 7 days postoperatively. The patient’s clinical symptoms and signs improved on the third day after nephrectomy. She was discharged on the seventh day postoperatively and has been followed up in our outpatient clinic without XGP recurrence. Her last recorded creatinine level was 1.3 mg/dL, and her estimated glomerular filtration rate was 52 mL/min/1.73 m^2^. Her father’s USG which was done at outpatient clinic revealed numerous cysts on both kidneys and liver. Genetic test for ADPKD could not be performed because her family did not consent.

## Discussion

This report presents the first documented case of XGP in a patient with PCKD, without known predisposing factors such as obstructive uropathy, recurrent UTIs, or a significant medical history. The diagnosis of PCKD was established for the first time during this hospitalization. CT played a crucial role in the diagnosis, as XGP presented a preoperative diagnostic challenge resembling cystic RCC, necessitating nephrectomy.

The etiology of XGP in adults remains largely unknown. However, most cases are associated with chronic urinary obstruction and infection. Urinary obstruction often occurs due to calculi, particularly staghorn stones, which are found in approximately 80% of patients. These calculi serve as a nidus for infection (1, 9). In addition, conditions leading to urinary obstruction, such as pyelo-ureteric junction obstruction, ureteropelvic duplication, ureteral schistosomiasis, and obstructing tumors (renal and transitional cell carcinomas), have been implicated (9). However, in our case, there was no radiologic evidence of urinary obstruction, and our patient denied experiencing recurrent UTIs. It is possible that the numerous cysts observed in our patient may have contributed to urinary flow obstruction, leading to asymptomatic recurrent bacterial superinfection and ultimately inducing XGP, although the exact mechanism remains unclear.

The exact pathophysiology of XGP remains unclear. It is presumed to involve a combination of obstruction and infection, leading to a chronic granulomatous immune response that fails to completely eradicate the inciting agent (10). This chronic inflammatory response, triggered by persistent infected debris, results in granuloma formation and destruction of renal parenchyma. The inability to clear bacterial products is associated with a limited or incomplete host immune response (11, 12). The pathogenesis of XGP involves various mechanisms, including alterations in immunologic response, changes in lipid metabolism, increased lymphatic blockage, and local vascular occlusion (13, 14). A recent study demonstrated that XGP specimens contain both classically activated macrophages (M1) and alternatively activated macrophages (M2), suggesting that the disease pathogenesis is related to repeated cycles of infection, inflammation, and attempted healing (15). Persistent obstructive uropathy perpetuates these cycles, leading to further inflammation and worsening of XGP until the renal parenchyma is extensively destroyed.

Symptoms of XGP depend on the disease severity and stage. Patients typically present with flank or abdominal pain, fever, dysuria, hematuria, a palpable mass, and weight loss. However, symptoms are often nonspecific, such as fatigue and general malaise, and may persist for more than 6 months in 42% of patients (9). Interestingly, urinary tract symptoms may be minimal or absent, leading to initial misdiagnosis. In some instances, the only symptoms may arise from extrarenal complications affecting organs such as the liver, spleen, chest wall, pleural space, abdominal wall, gluteus, and skin (1, 9). Patients with XGP often present in poor general condition due to the chronic nature of the disease, which can mimic malignancy or debilitating infections such as tuberculosis. Our patient initially experienced nonspecific symptoms of fatigue and general malaise without symptoms of UTIs.

Diagnosing XGP can be challenging due to its variable clinical presentation, ranging from asymptomatic radiological findings to more severe complications. It should be suspected in cases of recurrent UTIs occurring in the setting of chronic obstructive uropathy (1). CT is the primary imaging modality for its diagnosis, despite the possibility of overlapping imaging features with other conditions such as RCC, TCC, renal tuberculosis, and malakoplakia. The “bear-paw” sign on contrast-enhanced CT is a characteristic but not pathognomonic feature of XGP. However, a previous study demonstrated significant variation in imaging features in confirmed XGP cases (16), with a preoperative diagnosis accuracy of 28.2%. Another study reported that only 27.3% of patients are correctly diagnosed with XGP preoperatively (17). In one study, the preoperative suspected diagnosis rate of XGP using multidetector CT was 66.67% (18). Definitive diagnosis is typically established through histological examination of tissue specimens. In our patient, the preoperative suspicion was cystic RCC, leading to open nephrectomy due to lack of history of recurrent UTIs and the absence of urinary calculi on radiological imaging, despite XGP being suspected in CT exams. Notably, surgical intervention is often necessary in cases where there is no response to antibiotics.

XGP is staged according to the classification system proposed by Malek and Elder (19), which categorizes the disease into three stages based on the extent of surrounding tissue involvement. Stage I (nephric) typically involves only the nephric tissue, with some studies using the term focal disease to describe early-stage XGP where kidneys are partially affected and renal function remains partially preserved (1, 14, 16, 17). Stage II (perinephric) involves both the nephric and perinephric fatty tissues, often termed diffuse disease (1, 20, 21). Stage III (paranephric) involves the additional infiltration of paranephric tissues and organs, leading to complications with extrarenal involvement in surrounding organs (1, 22, 23). The classification of focal and diffuse diseases helps distinguish between different forms of XGP and facilitates potential management strategies. In addition, Goyal et al. (1) proposed a new category of extrarenal disease to highlight the unique clinical presentations and management challenges. Advancements in radiological imaging techniques have significantly helped delineate the extent of XGP and have helped guide treatment decisions. In our patient, the XGP was categorized as diffuse disease.

In the management of XGP, two primary therapeutic approaches are commonly employed, depending on the extent of disease: nephrectomy and antibiotics. Nephrectomy serves as the mainstay of XGP treatment due to the significant inflammation associated with the condition, which can obscure surgical planes and compromise renal function. Previously, nephrectomy was performed for nearly all cases (1–5, 24). The choice between partial and total nephrectomy, as well as between robotic/laparoscopic and open surgical approaches, remains controversial. Antibiotics and drainage alone are typically inadequate for focal XGP. Partial nephrectomy has shown limited success, with only 5% of cases reportedly being effectively treated using this approach (14). Salvaging viable renal tissue is challenging due to the extensive destruction typically observed in XGP, often resulting in obliteration of surgical planes. Nuclear medicine renal scans have revealed minimal renal function in the affected kidney in up to 80% of cases, rendering partial nephrectomy unnecessary (9). As a result, open total nephrectomy has been the preferred approach. While the use of laparoscopic nephrectomy for XGP is controversial, it may be considered in cases where an experienced surgeon is available and the inflammation is contained within Gerota’s fascia in focal and diffuse disease. However, Guzzo et al. (25) suggested that this is often challenging for XGP as it requires advanced surgical skills, and it should be offered only to highly selected patients.

Antibiotics should be utilized as adjunctive therapy in all XGP cases, as medical management alone is rarely sufficient to eradicate the infection (1). The selection of antibiotics depends on the organism cultured and antimicrobial susceptibility testing results. *E. coli* and *Proteus mirabilis* are the most commonly isolated pathogens, accounting for approximately 90% of positive urinary cultures in XGP patients, although sterile urine cultures are common (26). Studies have reported varying rates of positive urine cultures in XGP patients, ranging from 48.1 to 62.06% (26, 27). Empirical broad-spectrum antibiotics, such as extended-spectrum penicillin, third-generation cephalosporin, fluoroquinolone, and carbapenem, should be initiated until organism identification and antimicrobial susceptibility results are available. In our case, urine culture was sterile but tissue culture revealed *E. coli* susceptible to third-generation cephalosporin and fluoroquinolone.

In summary, XGP is a rare complication of pyelonephritis often associated with recurrent UTIs in the setting of chronic urologic obstruction. Its diagnosis is challenging as it can remain asymptomatic and its clinical and imaging findings may mimic other pathologies. Integrated analysis of clinical, laboratory, and imaging findings is crucial for accurate diagnosis. Physicians should be aware that the presence of numerous cysts in patients with PCKD may serve as a potential etiology for XGP, even in the absence of renal calculi and symptomatic UTIs. Early diagnosis and intervention are crucial for preserving kidney function and preventing life-threatening complications.

## Data availability statement

The raw data supporting the conclusions of this article will be made available by the authors, without undue reservation.

## Ethics statement

The study protocol was approved by the Institutional Review Board of Gyeongsang National University Changwon Hospital (IRB no. 2024-02-004). Written informed consent was obtained from the individual(s) for the publication of any potentially identifiable images or data included in this article. Written informed consent was obtained from the participant/patient(s) for the publication of this case report.

## Author contributions

YK: Conceptualization, Writing – original draft, Validation. TL: Investigation, Resources, Writing – original draft. EB: Conceptualization, Methodology, Resources, Writing – review & editing. HJ: Conceptualization, Investigation, Resources, Writing – review & editing. SJ: Conceptualization, Resources, Validation, Writing – review & editing. SL: Conceptualization, Methodology, Resources, Writing – review & editing. S-HC: Investigation, Supervision, Validation, Writing – review & editing. DP: Conceptualization, Supervision, Writing – review & editing.
